# Nafamostat and sepimostat identified as novel neuroprotective agents via NR2B N-methyl-D-aspartate receptor antagonism using a rat retinal excitotoxicity model

**DOI:** 10.1038/s41598-019-56905-x

**Published:** 2019-12-31

**Authors:** Masahiro Fuwa, Masaaki Kageyama, Koji Ohashi, Masaaki Sasaoka, Ryuichi Sato, Masami Tanaka, Kei Tashiro

**Affiliations:** 1 Research and Development, Santen Pharmaceutical Co., Ltd, Nara, Japan; 2 Global Alliances and External Research, Santen Pharmaceutical Co., Ltd, Nara, Japan; 3 Department of Genomic Medical Sciences, Kyoto Prefectural University of Medicine, Kyoto, Japan

**Keywords:** Neuroscience, Neurodegeneration, Eye diseases, Drug development, Receptor pharmacology

## Abstract

In addition to its role in the treatment of pancreatitis, the serine protease inhibitor nafamostat exhibits a retinal protective effect. However, the exact mechanisms underlying this effect are unknown. In this study, the neuroprotective effects of nafamostat and its orally active derivative sepimostat against excitotoxicity were further characterised *in vitro* and *in vivo*. In primary rat cortical neurons, nafamostat completely suppressed N-methyl-D-aspartate (NMDA)-induced cell death. Intravitreal injection of nafamostat and sepimostat protected the rat retina against NMDA-induced degeneration, whereas the structurally related compounds, gabexate and camostat, did not. The neuroprotective effects of nafamostat and the NR2B antagonist ifenprodil were remarkably suppressed by spermidine, a naturally occurring polyamine that modulates the NR2B subunit. Both nafamostat and sepimostat inhibited [^3^H]ifenprodil binding to fractionated rat brain membranes. Thus, nafamostat and sepimostat may exert neuroprotective effects against excitotoxic retinal degeneration through NMDA receptor antagonism at the ifenprodil-binding site of the NR2B subunit.

## Introduction

Nafamostat mesilate (nafamostat) is a synthetic serine protease inhibitor routinely used for the treatment of acute pancreatitis in Japan^1,2^. Nafamostat has potent inhibitory effects on multiple types of serine proteases including trypsin, thrombin, plasmin and complement components^3^. Although this small molecular compound was synthesised in the early 80s, its pharmacological profiles are still being investigated. These efforts have led to the discovery of novel mechanisms of action and potential alternative indications such as brain^4^ and kidney ischaemic injury^5^, spinal cord injury^6^ and cancer^7,8^. Particularly, it is worth noting that nafamostat improved locomotion activities and reduced tissue damage following spinal cord injury in rats in a previous, study^6^ suggesting that the functional and morphological improvements induced by nafamostat is due to reduced apoptosis through the suppression of proinflammatory cytokine production and increased expression of neurotrophins along with decreased expression of thrombin, a targeted protein for its authentic pharmacological effects. Furthermore, another study^9^ demonstrated that nafamostat preserved neuronal axons and dendrites in a chronic ischaemic stroke model suggesting that nafamostat promotes axonal regeneration. Collectively, nafamostat seems to have great potential as a neuroprotectant for the treatment of neurodegenerative diseases.

Glaucoma is an ocular neurodegenerative disease characterised by predominant retinal ganglion cell (RGC) loss followed by progressive visual field defects and is the leading cause of irreversible blindness worldwide^10–13^. Although the aetiology of glaucoma remains largely unknown, RGC loss may stem from apoptosis triggered by genetic and environmental factors including the loss of support from neurotrophic factors, oxidative stress, neuroinflammation and excitotoxicity^14–18^. To repurpose clinically and regulatory approved drugs as neuroprotectants for glaucoma, we screened a wide variety of compounds including nafamostat using primary rat cortical neurons and an N-methyl-D-aspartate (NMDA)-induced retinal degeneration rat model. Among the screened compounds, nafamostat was one of the most potent and promising candidates for neuroprotection. Furthermore, we identified sepimostat, a structurally related compound with improved oral bioavailability^19,20^, as a neuroprotectant equivalent to nafamostat. Because the safety profiles of these compounds have been well characterised in clinical use, it was considered worthwhile to evaluate them further as a potential new class of neuroprotective drugs. Although a similar retinal protective effect of nafamostat was already reported elsewhere^21^, the exact molecular mechanisms of its action have not been addressed and remain unclear.

The present study examines the neuroprotective effects and modes of action of nafamostat and sepimostat against retinal degeneration induced by NMDA, kainate and ischaemia/reperfusion. Here we report that both nafamostat and sepimostat have significant neuroprotective effects against excitotoxicity-mediated retinal degeneration. We also propose that their neuroprotective effects may be mediated primarily by NMDA receptor antagonism at the ifenprodil-binding site of the NR2B subunit, irrespective of their original pharmacological actions as serine protease inhibitors.

## Results

### Neuroprotective effects of nafamostat on NMDA-induced neuronal cell death *in vitro*

**Figure 1:**
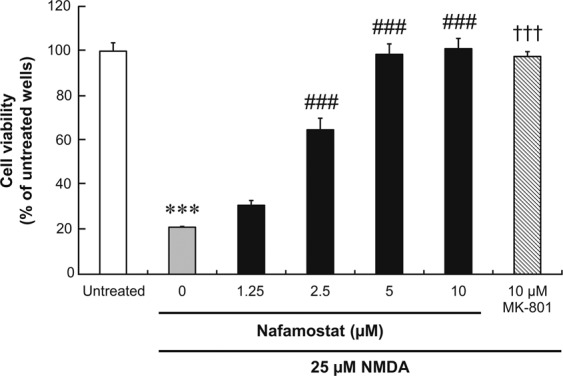
The concentration–response relationship for the neuroprotective effects of nafamostat against NMDA-induced cell death in primary rat cortical neurons. Neurons were incubated for 2 h simultaneously with nafamostat and NMDA, and cell viability was assessed using MTS assays. Absorbance in each well was normalised to that in the untreated wells (Untreated; medium alone) and presented as percentages. Each value represents the mean ± S.E. of six replicates. ^***^P < 0.001, compared with untreated control; ^†††^P < 0.001, compared with NMDA alone by Aspin–Welch’s t-test. ^###^P < 0.001, compared with NMDA alone by Dunnett’s multiple comparison test.

To determine whether nafamostat has neuroprotective effects, we first examined its effects on NMDA-induced neuronal cell death in cultured primary rat cortical neurons. Figure 1 shows the concentration–response curves for the effects of nafamostat on NMDA-induced neuronal cell death in comparison with those of MK-801, an authentic NMDA receptor antagonist used as the positive control. In the absence of either nafamostat or MK-801, the application of 25 µM NMDA to the culture medium resulted in 80% reduction in the cell viability, which reached statistical significance. Nafamostat demonstrated a potent and concentration-dependent neuroprotective effect against NMDA-induced neuronal cell death. This effect was statistically significant in the range from 2.5 to 10 µM and reached the peak at 5 µM. As expected, 10 µM MK-801 also provided statistically significant and complete protection against NMDA-induced neuronal cell death. Thus, nafamostat may have an *in vitro* neuroprotective effect equivalent to that of MK-801, which has been assessed clinically^22^.

### Neuroprotective effects of nafamostat and sepimostat on NMDA- and ischaemia/reperfusion-induced retinal degeneration

**Figure 2:**
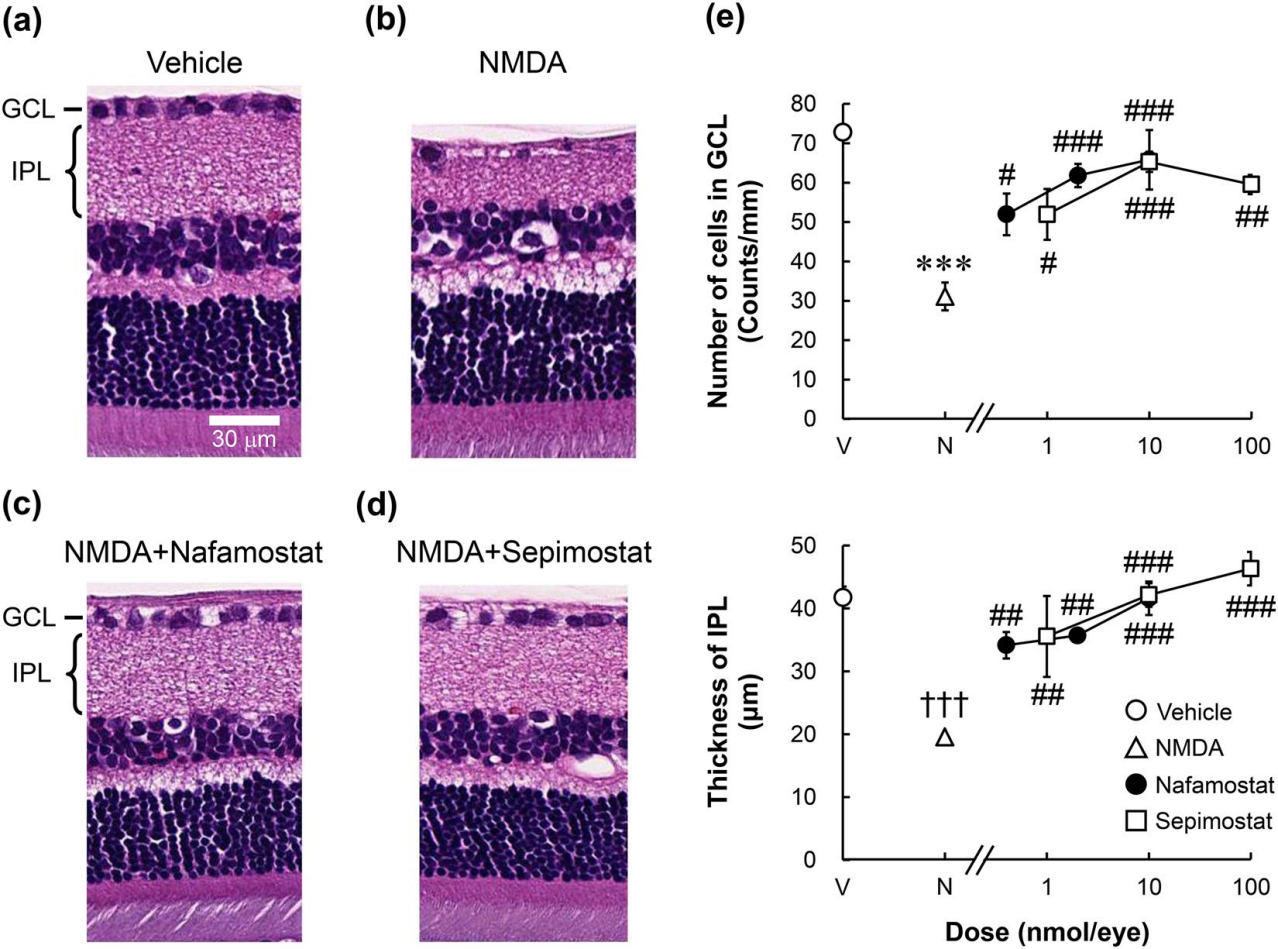
Effects of nafamostat and sepimostat on NMDA-induced retinal degeneration in rats. Panels a–d show the typical histological appearance of the retinas after intravitreal injections of vehicle (**a**), NMDA alone (**b**, 20 nmol/eye), NMDA plus nafamostat (**c**, 2 nmol/eye), or sepimostat (**d**, 10 nmol/eye). The scale bar in panel a represents 30 µm. GCL: ganglion cell layer; IPL: inner plexiform layer. Panel e shows the dose–response curves for the protective effects of nafamostat (closed circle, 0.4, 2 and 10 nmol/eye) and sepimostat (open square, 1, 10 and 100 nmol/eye) against NMDA-induced retinal degeneration. The vehicle (V) and NMDA (N) are shown as an open circle and open triangle, respectively. The upper panel shows the GCL cell number, and the lower panel shows the IPL thickness. Each value represents the mean ± S.E. for four to six rats. ^***^P < 0.001, compared with vehicle by Student’s t-test. ^†††^P < 0.001, compared with vehicle by Aspin–Welch’s t-test. ^#^P < 0.05, ^##^P < 0.01, ^###^P < 0.001, compared with NMDA alone by Dunnett’s multiple comparison test.

To further characterise the neuroprotective effects of nafamostat and its derivative sepimostat *in vivo*, we first examined their effects on retinal degeneration induced by NMDA and ischaemia in rats. Figure 2a–d show the typical histological appearance of rat retinas 2 weeks after intravitreal injection of the vehicle and 20 nmol/eye NMDA, with or without concomitant injections of nafamostat and sepimostat. A decreased cell count in the ganglion cell layer (GCL) and reduced thickness of the inner plexiform layer (IPL) were observed after NMDA injection (Fig. 2b). These changes are hallmarks of NMDA-induced retinal degeneration as reported in many studies^23,24^. Intravitreal injection of either 2 nmol/eye nafamostat (Fig. 2c) or 10 nmol/eye sepimostat (Fig. 2d) simultaneously with NMDA completely prevented NMDA-induced changes in retinal morphology. As shown in Fig. 2e, the neuroprotective effects of nafamostat and sepimostat were statistically significant and dose-dependent in the range from 0.4 to 10 nmol/eye and 1 to 100 nmol/eye, respectively.

**Figure 3:**
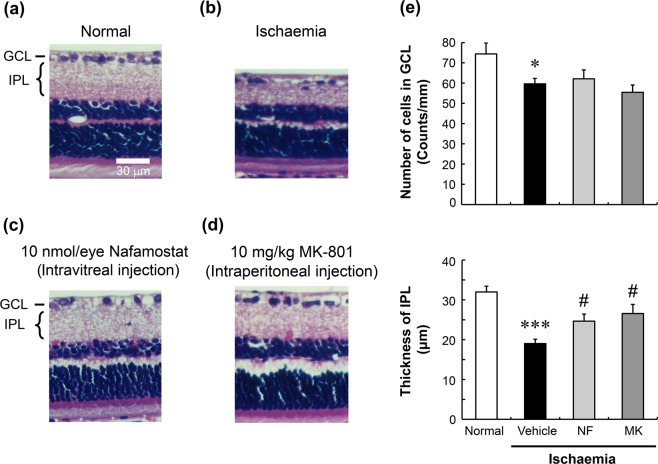
Effects of nafamostat on ischaemia/reperfusion-induced retinal degeneration in rats. Panels a–d show the typical histological appearance of retinas before (normal, **a**) and after ischaemia/reperfusion, the latter with intravitreal injections of vehicle (ischaemia, **b**) and nafamostat (**c**, 10 nmol/eye), and with intraperitoneal injection of MK-801 (**d**, 10 mg/kg). The scale bar in panel equals 30 µm. Panel e shows the quantitative results for the protective effects of nafamostat (NF, light-grey column) and MK-801 (MK, dark-grey column) on ischaemia-induced retinal degeneration. Normal control and ischaemia alone are shown in open and closed columns, respectively. The upper panel shows the GCL cell number, and the lower panel shows the IPL thickness. Each value represents the mean ± S.E. for four to seven rats. ^*^P < 0.05; ^***^P < 0.001, compared with normal control by Student’s t-test. ^#^P < 0.05, compared with ischaemia alone by Dunnett’s multiple comparison test.

Figure 3 shows the effects of nafamostat and MK-801 on retinal degeneration induced by ischaemia/reperfusion. Forty-five minutes following high intraocular pressure-induced retinal ischaemia, the animals were allowed to recover for a week (reperfusion). Ischaemia/reperfusion reduced not only the GCL cell number but also the IPL thickness (Fig. 3b,e). Intravitreal injection of 10 nmol/eye nafamostat 1 h prior to ischaemic injury significantly inhibited the reduction in IPL thickness caused by retinal ischaemia/reperfusion but did not correct the loss of cells in the GCL (Fig. 3c,e). Similarly, intraperitoneal injection of 10 mg/kg MK-801 1 h prior to ischaemia ameliorated the reduced thickness of the IPL in this model, without affecting the loss of cells in the GCL (Fig. 3d,e).

### Reversal of the neuroprotective effect of nafamostat by spermidine

**Figure 4:**
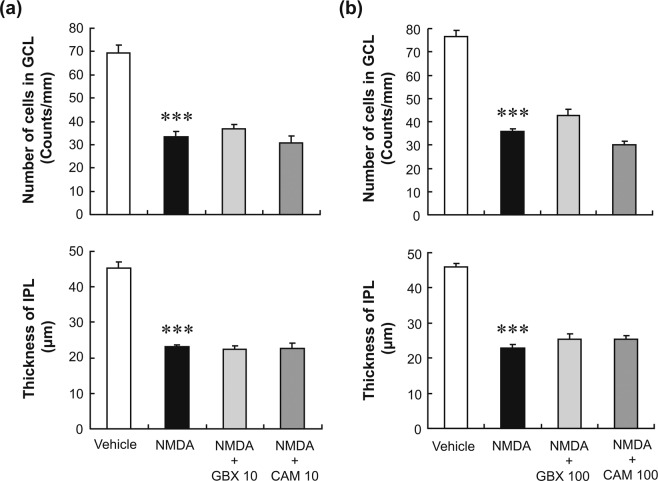
Effects of gabexate and camostat on NMDA-induced retinal degeneration in rats. Panel a shows the changes in the GCL cell number (upper panel) and IPL thickness (lower panel) after intravitreal injections of vehicle (open column), NMDA alone (closed column, 20 nmol/eye), NMDA plus gabexate (GBX, light-grey column, 10 nmol/eye), or camostat (CAM, dark-grey column, 10 nmol/eye). Panel b is shown in the same manner as in (**a**), except that a higher concentration (100 nmol/eye) of either gabexate or camostat was given to the animals. Each value represents the mean ± S.E. for three to five rats. ^***^P < 0.001, compared with vehicle by Student’s t-test.

Because the best-characterised pharmacological effects of nafamostat and sepimostat are the inhibition of serine protease activities^25^, we questioned whether this inhibition might be involved in the neuroprotective effects of the two compounds. To test this possibility, we examined the effects of gabexate and camostat, which are serine protease inhibitors with chemical structures closely related to those of nafamostat and sepimostat (see Supplementary Fig. S1), on NMDA-induced retinal degeneration. Intravitreal injection of either 10 nmol/eye gabexate or camostat did not affect the retinal degeneration induced by NMDA (Fig. 4a). Furthermore, these protease inhibitors had no effect on NMDA-induced retinal degeneration even at the highest dose tested, 100 nmol/eye (Fig. 4b). This dose would have resulted in intravitreal concentrations of approximately 1.7 mM, as the volume of the vitreous body of rats is assumed to be 60 µL^26^. In fact, 1.7 mM is much higher than the concentration commonly used to inhibit serine protease activities^25^. These results clearly indicate that the inhibition of serine protease activities by nafamostat and sepimostat play no role in their neuroprotective effects against NMDA-induced retinal degeneration.

**Figure 5:**
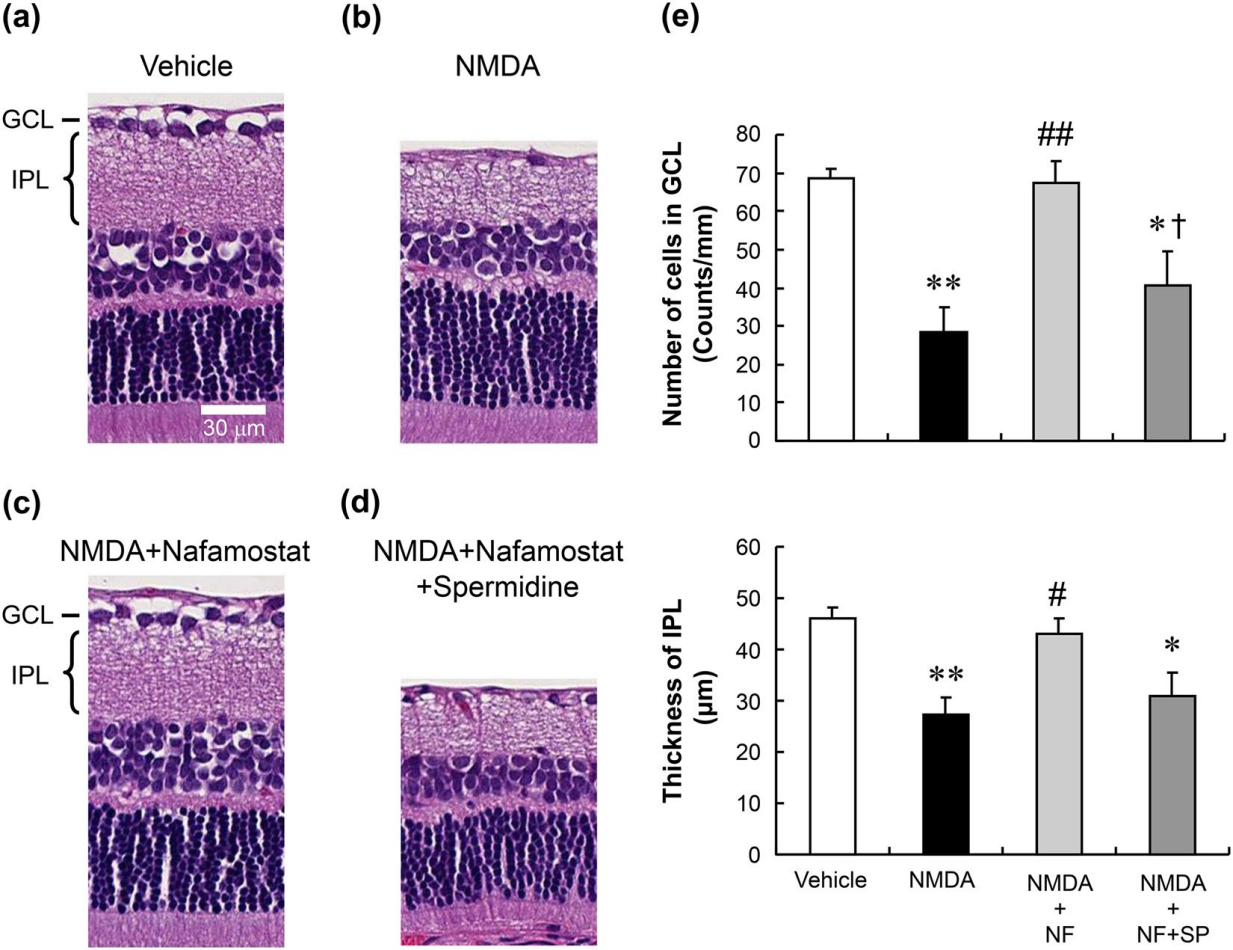
Reversal by spermidine of the neuroprotective effect of nafamostat. Panels a–d show the typical histological appearance of rat retinas after intravitreal injections of vehicle (**a**), NMDA alone (**b**, 20 nmol/eye), NMDA plus nafamostat (**c**, 10 nmol/eye) and NMDA with nafamostat plus spermidine (**d**, 50 nmol/eye). The scale bar equals 30 µm. Panel e shows the changes in the GCL cell number (upper) and IPL thickness (lower) after intravitreal injections of vehicle (open column), NMDA alone (closed column, 20 nmol/eye), NMDA plus nafamostat (NF, light-grey column, 10 nmol/eye) and NMDA plus nafamostat and spermidine (SP, dark-grey column, 50 nmol/eye). Each value represents the mean ± S.E. for four to five rats. ^*^P < 0.05; ^**^P < 0.01, compared with vehicle, ^#^P < 0.05; ^##^P < 0.01, compared with NMDA alone and ^†^P < 0.05, compared with NMDA plus NF by Tukey’s multiple comparison test.

We considered the alternative possibility that NMDA receptor antagonism might mediate the neuroprotective effects of nafamostat and sepimostat. This is based on the fact that they possess an amidinophenyl group, a feature shared with pentamidine (see Supplementary Fig. S1), which is an antimicrobial agent with an NMDA receptor antagonistic property^27^. Because one facet of NMDA receptor antagonism by pentamidine is the reversal of such activity by polyamines like spermidine^27^, we tested the ability of spermidine to modify the neuroprotective effects of nafamostat against NMDA-induced retinal degeneration. Figure 5a–d show representative images illustrating the neuroprotective efficacy of 10 nmol/eye nafamostat in the presence of 20 nmol/eye NMDA, with and without 50 nmol/eye spermidine. Again, intravitreal injection of nafamostat completely inhibited NMDA-induced retinal degeneration (Fig. 5c). The neuroprotective effect of nafamostat was reversed when injected together with spermidine (Fig. 5d). The reversal of the protective effect of nafamostat against the NMDA-induced loss of cells in the GCL by spermidine was statistically significant, although the reversal with respect to protection against the NMDA-reduced IPL thickness was not statistically significant (Fig. 5e).

**Figure 6:**
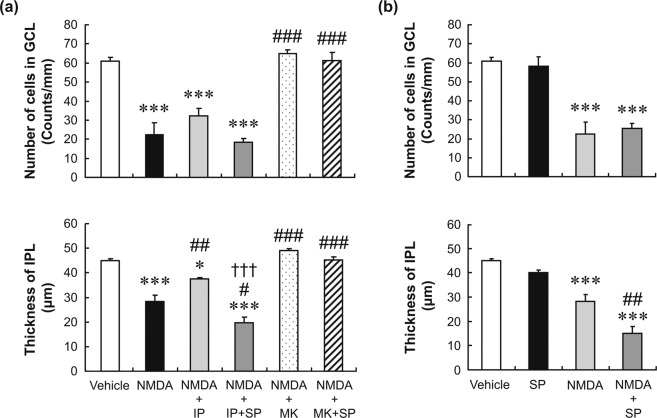
Reversal by spermidine of the neuroprotective effect of ifenprodil, but not that of MK-801, on rat retinas exposed to NMDA. Panel a shows the changes in the GCL cell number (upper) and the IPL thickness (lower) after intravitreal injections of vehicle (open column), NMDA alone (closed column, 20 nmol/eye), NMDA plus ifenprodil (IP, light-grey column, 10 nmol/eye), NMDA plus ifenprodil and spermidine (SP, dark-grey column, 50 nmol/eye), NMDA plus MK-801 (MK, dotted column, 10 nmol/eye) and NMDA plus MK-801 and spermidine (cross-hatched column). Panel b shows the changes in the GCL cell number (upper) and the IPL thickness (lower) after intravitreal injections of vehicle (open column), spermidine alone (SP, closed column, 50 nmol/eye), NMDA alone (light-grey column, 20 nmol/eye) and NMDA plus spermidine (dark-grey column). Each value represents the mean ± S.E. for four to five rats. ^*^P < 0.05; ^***^P < 0.001, compared with vehicle, ^#^P < 0.05; ^##^P < 0.01; ^###^P < 0.001, compared with NMDA alone and ^†††^P < 0.001, compared with NMDA plus ifenprodil (all by Tukey’s multiple comparison test).

To confirm the mechanistic specificity of the reversal of the neuroprotective effect of nafamostat by spermidine, we examined the effects of spermidine on the neuroprotective efficacies of ifenprodil and MK-801 in the NMDA-induced retinal degeneration model. Ifenprodil is known to bind specifically to the NR2B subunit of NMDA receptors (see Supplementary Fig. S2)^28^, and its neuroprotective effect was also reported to be suppressed by spermidine^29^. In contrast, MK-801 binds to the site within the channel pore of the NR1/NR2 receptor complex (see Supplementary Fig. S2)^30^, and its effect does not appear to be modified by spermidine^31^. As shown in Fig. 6a, intravitreal injection of ifenprodil (10 nmol/eye) significantly inhibited the reduction in IPL thickness induced by simultaneous injection of NMDA, but not the decrease in the GCL cell number. MK-801 (10 nmol/eye) completely inhibited the NMDA effects in both the GCL and the IPL. Not surprisingly, prevention of NMDA-induced retinal degeneration by ifenprodil was markedly suppressed by the simultaneous injection of spermidine, whereas neuroprotection by MK-801 was not affected. Spermidine exposure alone had little effect on the GCL cell number or IPL thickness but slightly potentiated the reduction of IPL thickness in the presence of NMDA, in the absence of other agents (Fig. 6b). These results suggest that the neuroprotective effect of nafamostat is mediated by the antagonism of NMDA receptors, probably at the polyamine site and/or the ifenprodil-binding site in the NR2B subunit.

### Effect of nafamostat on [^3^H]ifenprodil binding

**Figure 7:**
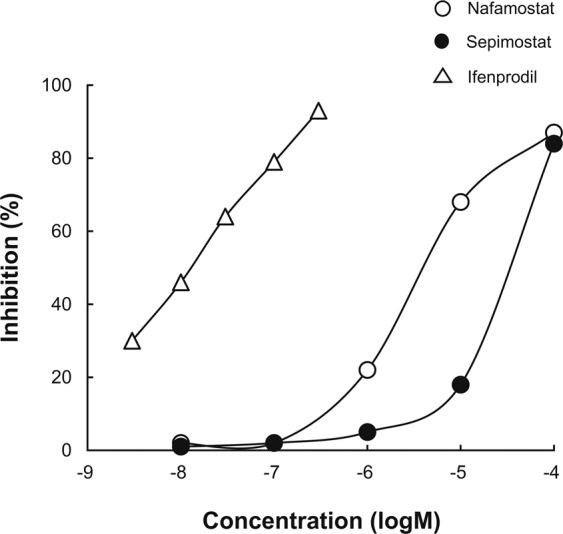
Effects of nafamostat and sepimostat on [^3^H]ifenprodil-binding in rat cerebral cortical membranes. Binding assay was performed in the presence of 5 µM GBR 12909, a sigma receptor antagonist. Open and closed circles show the percentages of [^3^H]ifenprodil binding inhibition by nafamostat and sepimostat, respectively, and open triangle shows the same using unlabelled ifenprodil as a positive control. The IC_50_ values for nafamostat, sepimostat and ifenprodil were 4.52, 29.8, and 0.0121 µM, K_i_ values were 4.20, 27.7 and 0.0112 µM, and Hill coefficients were 0.816, 1.32 and 0.664, respectively. Each value represents the mean of two to three replicates.

To determine whether nafamostat and sepimostat directly interact with the ifenprodil-binding site in the NMDA receptor complex, we performed receptor-binding assays using [^3^H]ifenprodil with rat cerebral cortex membranes. Figure 7 shows the displacement curves for the [^3^H]ifenprodil binding by nafamostat and sepimostat in comparison with unlabelled ifenprodil. Unlabelled ifenprodil inhibited the [^3^H]ifenprodil binding in a monophasic manner with a K_i_ value of 0.0112 µM and a Hill coefficient of 0.664. Nafamostat and sepimostat also inhibited the ifenprodil binding with K_i_ values of 4.20 and 27.7 µM, respectively. The Hill coefficients for nafamostat and sepimostat were 0.816 and 1.32, respectively, which were effectively close to unity. The concentrations of nafamostat and sepimostat in the vitreous bodies of eyes injected with 2 and 10 nmol/eye (corresponding to the doses that produced their respective maximum neuroprotective effects) would reach approximately 33 and 170 µM, respectively (the same vitreous volume assumption as above). These concentrations are 6 to 8 times higher than the K_i_ values for the inhibition of ifenprodil binding by nafamostat and sepimostat, suggesting that their concentrations after intravitreal injections reach levels substantially enough to inhibit ifenprodil binding. As a result, both nafamostat and sepimostat may be expected to bind to the ifenprodil-binding site in the NMDA receptor complex, most likely to the NR2B subunit.

### Effect of nafamostat on kainate-induced retinal degeneration

**Figure 8:**
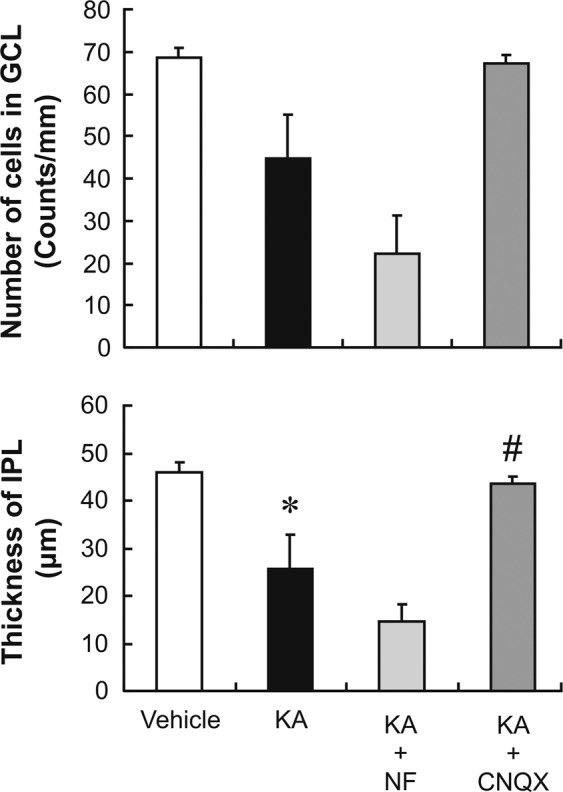
Effects of nafamostat and CNQX on kainate-induced retinal degeneration in rats. Upper and lower panels show the changes in the GCL cell number and IPL thickness, respectively, after intravitreal injections of the vehicle (open column), kainate alone (KA, closed column, 5 nmol/eye), kainate plus nafamostat (NF, light-grey column, 10 nmol/eye), or cyanquixaline (CNQX, dark-grey column, 5 nmol/eye). Each value represents the mean ± S.E. for four to five rats. ^*^P < 0.05, compared with vehicle by Student’s t-test. ^#^P < 0.05, compared with KA alone by Dunnett’s multiple comparison test.

To further determine whether nafamostat exerts its neuroprotective effect through non-NMDA receptor antagonism, we examined its effect on kainate-induced retinal degeneration. Figure 8 shows the effects of kainate on the GCL and the IPL in the presence or absence of nafamostat or cyanquixaline (CNQX), an AMPA/kainate receptor antagonist. Intravitreal injection of kainate (5 nmol/eye) caused a mild, but statistically significant, reduction in IPL thickness. Kainate also caused a decrease in GCL cell number, but this change did not attain statistical significance. The kainate-induced retinal degeneration was completely inhibited by the simultaneous injection of CNQX with kainate. However, nafamostat had no effect on the kainate-induced changes in either the GCL or the IPL.

## Discussion

The present study determined that nafamostat and its derivative sepimostat had potent neuroprotective effects *in vitro* and *in vivo*: (1) Nafamostat protected cortical neurons against NMDA-induced neuronal cell death; (2) it ameliorated the retinal degeneration induced by ischaemia/reperfusion; and (3) both compounds completely suppressed NMDA-induced retinal degeneration. Surprisingly, the inhibition of serine protease activity may not underlie the neuroprotective efficacy of the two compounds we tested, despite the fact that the best understood primary molecular targets for both nafamostat and sepimostat are serine proteases, including trypsin, thrombin and plasmin^3,20^. Instead, it is more likely that specific antagonism of NMDA receptors through binding to the NR2B subunit mediates the neuroprotective effects of nafamostat and sepimostat, similar to what is seen with ifenprodil. Nafamostat and sepimostat were discovered over 30 years ago and have since been clinically applied with no serious adverse effects in Japan^1,2^. This study is the first report on their NMDA receptor antagonism at the NR2B subunit in the long history of their research and development, and the results were substantiated by a receptor-binding assay.

The current study significantly extends our understanding of the neuroprotective effect of nafamostat and its mode of action, which were first reported by Tsuda *et al*^[.21^. Given serine protease inhibition as the primary pharmacological effect, they unexpectedly found that nafamostat increased tryptase-like protease activity in the retina in the absence and presence of NMDA. To address this puzzle, we took a different and simpler approach using the structurally related compounds, gabexate and camostat, which have very similar pharmacological profiles to those of nafamostat and sepimostat^32–34^. We found that unlike nafamostat and sepimostat, both gabexate and camostat failed to show neuroprotective effects against NMDA-induced retinal degeneration, suggesting no role for serine protease inhibition in the neuroprotective effects of either nafamostat or sepimostat. Our finding is inconsistent with those of earlier studies showing that genetic knockout of tissue plasminogen activator inhibited NMDA-induced retinal apoptosis^35^ and that plasminogen activator inhibitor had the same effect on a kainate-induced retinal degeneration model^36^. Specifically, tissue plasminogen activator converts plasminogen into plasmin, which can activate matrix metalloproteinases, leading to extracellular matrix destruction and RGC death^37^. However, it is unlikely that nafamostat and sepimostat exert their neuroprotective effects through plasmin inhibition, because aprotinin, a naturally occurring serine protease inhibitory polypeptide, also failed to suppress NMDA-induced retinal degeneration (see Supplementary Fig. S3). Therefore, serine protease inhibition plays only a minor role, if any, in NMDA-induced retinal degeneration and the neuroprotective effects of nafamostat and sepimostat.

The finding that nafamostat showed specificity for NMDA-induced as opposed to kainite-induced retinal degeneration suggests that its neuroprotective effect is mediated through interaction with NMDA receptors, not AMPA/kainate receptors. Using the PubChem database developed by the National Center for Biotechnology Information, we searched for a chemical structural similarity between nafamostat derivatives and currently known NMDA antagonists. This resulted in the identification of the amidinophenyl group as a structure shared by nafamostat and pentamidine, an antimicrobial compound with an NMDA receptor antagonistic property^27^. An NMDA receptor-binding study on pentamidine showed that it inhibits MK-801-specific binding in a concentration-dependent manner and that its inhibitory effect on MK-801 binding was reduced in the presence of spermidine, a naturally occurring polyamine^27^. Whether pentamidine has a neuroprotective effect in the retina and whether this effect is modified by spermidine is unknown at present. However, it was reported that spermidine suppressed the neuroprotective effect of ifenprodil in rat cultured retinal neurons^29^. The inhibitory effect of ifenprodil on MK-801 binding was also attenuated by spermidine^38^, as also seen with pentamidine. In this study, we found that spermidine markedly reduced the neuroprotective effects of nafamostat and ifenprodil, whereas it had no effect on that of MK-801. These results suggest that like ifenprodil, nafamostat produces a neuroprotective effect in the retina through the antagonism of NMDA receptors at the polyamine site and/or the site(s) close to it.

**Figure 9:**
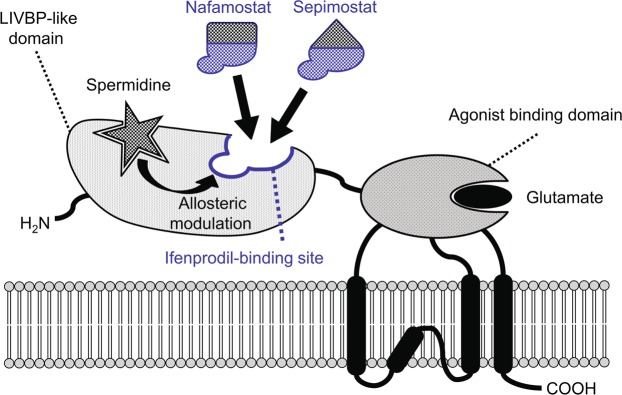
Schematic diagram of the protein structure of the NMDA receptor NR2B subunit and a potential binding site of nafamostat and sepimostat. The original drawings provided by Perin-Dureau *et al*.^42^ and Marinelli *et al*.^43^ are modified. The ifenprodil-binding site is located in the extracellular region of the NR2B subunit designated as the N-terminal leucine/isoleucine/valine-binding protein (LIVBP)-like domain. Spermidine binds to the site near the ifenprodil-binding site of the LIVBP and allosterically modulates the ifenprodil binding. Nafamostat and sepimostat may competitively bind to the ifenprodil-binding site, and their binding may be allosterically modified by spermidine.

NMDA receptors consist of heterooligomers of NR1 subunits and one or more of four NR2 subunits, which are designated as NR2A-D^39,40^. Among these NR2 subunits, NR2B is the ifenprodil-binding site^41^. Figure 9 depicts the protein structure of the NR2B subunit and the potential binding sites of the ligands used in this study. A fine mapping of the NR2B subunit using chimeras of NR2A and NR2B and point mutations of NR2B^42,43^ showed that ifenprodil binds to the N-terminal leucine/isoleucine/valine-binding protein (LIVBP)-like domain, which is located in the first 380 amino acid residues of the extracellular region of NR2B and has a structural similarity with bacterial periplasmic-binding protein. The ifenprodil-binding site is different from the polyamine-binding site, but there is an allosteric and non-competitive interaction between these two sites^44^. Similarly, the pentamidine-binding site is also known to be allosterically modified by polyamines, although its exact location in NMDA receptors is unclear^27^. The ifenprodil-binding assay in the present study showed that both nafamostat and sepimostat competitively inhibited [^3^H]ifenprodil binding in rat brain membranes with Hill coefficients close to unity. On the other hand, pentamidine also inhibited ifenprodil binding, but the Hill coefficient was much higher than unity (see Supplementary Fig. S4). These results suggest that even though the chemical structures of nafamostat and sepimostat differ from that of ifenprodil, they bind to the ifenprodil-binding site of the LIVBP-like domain, whereas pentamidine binds to other sites. An analysis of the structure–activity relationship of several ifenprodil derivatives revealed that three ifenprodil–NR2B interacting sites, which consist of a single hydrophobic binding site for the benzyl ring, a hydrogen bond donor/acceptor site for the central nitrogen atom and a hydrophobic and electrostatic binding site for the phenyl ring, are necessary for ifenprodil binding to the NR2B subunit^45,46^. If these chemical properties of ifenprodil are shared by nafamostat and sepimostat, this may be one reason why nafamostat and sepimostat recognise the ifenprodil-binding site, despite their different chemical structures. A detailed characterisation of the NMDA receptor-binding properties of nafamostat and sepimostat using molecular biological techniques and docking simulation would be desirable to delineate precisely the locations of their binding sites in NMDA receptors.

The clinical development of prototypical NMDA receptor antagonists including MK-801 has been mostly unsuccessful because of serious central nervous system (CNS) side effects such as hallucination and memory impairment^22^. Compared with prototypical NMDA receptor antagonists, subtype-selective antagonists like NR2B ligands are believed to have better CNS safety profiles^41^. Since the ifenprodil-binding site was identified in the NR2B subunit, many types of ifenprodil derivatives have been synthesised, and their feasibilities as neuroprotective drugs have been evaluated in animals^47–50^. These studies have shown that even at doses producing maximum neuroprotective effects, these NR2B antagonists have considerably fewer CNS side effects than MK-801. On the basis of such promising preclinical results, some of the NR2B antagonists have been clinically evaluated. However, they did not display clinical efficacy and/or had unexpected cardiovascular side effects^22,51^. Because nafamostat and sepimostat have novel chemical structures as well as different physicochemical and pharmacological properties from the ifenprodil derivatives, modification of their chemical structures may be an alternative approach to develop better NR2B antagonists for clinical use as well as useful pharmacological tools for further elucidation of NR2B biology. Although nafamostat is a very potent serine protease inhibitor, it can be used for this therapeutic purpose only through intravenous injection, not orally, because of its poor oral bioavailability^3^. To improve its pharmacokinetic profiles, various derivatives have been synthesised, and sepimostat was found to have much higher oral activity^19^. Sepimostat administered orally has been clinically evaluated for chronic pancreatitis and gastroesophageal reflux disease after stomach surgery and showed good safety profiles in clinical trials^52,53^. Therefore, it seems worthwhile to evaluate whether sepimostat might slow the progression of the visual field defect in glaucoma patients and also whether it can also be applied to other neurodegenerative diseases, even though its clinical development was terminated for unknown reasons.

In summary, the present study demonstrated that nafamostat and sepimostat are neuroprotective against NMDA- and ischaemic/reperfusion-induced retinal degeneration in rats. Furthermore, we found that these neuroprotective effects may be mediated by NMDA receptor antagonism, most likely via interactions at the ifenprodil-binding site of the NR2B subunit. Further studies are underway to determine the effects of nafamostat and sepimostat on retinal degeneration in different animal models and the exact locations of their binding sites in NMDA receptors.

## Methods

### Chemicals

Nafamostat mesilate (Futhan™) was purchased from Torii Pharmaceutical Co., Ltd. (Osaka, Japan) for *in vivo* study and Tokyo Chemical Industry Co., Ltd. (Tokyo, Japan) for *in vitro* study. Sepimostat mesilate was synthesised at NARD Institute Ltd. (Hyogo, Japan). Gabexate mesilate (FOY™) and camostat mesilate were obtained from Ono Pharmaceutical Co., Ltd. (Osaka, Japan) and Wako Pure Chemical Industries, Ltd. (Osaka, Japan). The chemical structures of these protease inhibitors are shown in Supplementary Fig. S1. NMDA, kainate, MK-801 and CNQX were obtained from Sigma (Saint Louis, MO, USA). Ifenprodil hemitartrate and spermidine trihydrochloride were obtained from Tocris (Ellisville, MO, USA) and Calbiochem (La Jolla, CA, USA).

### *In vitro* cell viability assay

Cortical neurons were prepared from 16-day-old Sprague Dawley rat embryos (Charles River Laboratories Japan, Inc., Yokohama, Japan). Cortices were dissociated using the Papain Dissociation System (Worthington Biochemical Corporation, Lakewood, NJ, USA) according to the manufacturer’s instructions, and the cells were seeded onto 96-well plates pre-coated with poly-L-lysine (AGC Techno Glass Co., Ltd., Shizuoka, Japan) at a cell density of 5.0 × 10^4^ cells/well. The cells were placed in a 5% CO_2_ incubator at 37°C and cultured for 20 days in the Neurobasal Plus Medium supplemented with 2% B27 Plus Supplement and 40 μg/mL gentamicin (all from Thermo Fisher Scientific Inc., Waltham, MA, USA). The culture medium was exchanged every 3–4 days and removed just before the application of NMDA. The cells were simultaneously incubated for 2 h with the test compounds and NMDA, and the cell viability was assessed using 3-(4,5-dimethylthiazol-2-yl)-5-(3-carboxymethoxyphenyl)-2-(4-sulfophenyl)-2H-tetrazolium (MTS) assays in accordance with the manufacturer’s instructions (CellTiter 96 AQ_ueous_ One Solution Cell Proliferation Assay, Promega Inc., Madison, WI, USA).

### Experimental animals

All the experimental procedures and animal care were performed in compliance with the ARVO Statement for the Use of Animals in Ophthalmic and Vision Research, with the necessary approval and monitoring by the Animal Care and Use Committee at Santen Pharmaceutical Co., Ltd.

Male Sprague Dawley rats (Charles River Laboratories Japan, Inc., Yokohama, Japan) weighing 150–300 g were anaesthetised by inhalation of 3% halothane and maintained with 1% halothane in 70% N_2_O and 30% O_2_. After pupil dilatation with a topical application of tropicamide and phenylephrine hydrochloride (Mydrin^®^-P, Santen Pharmaceutical Ltd., Osaka, Japan), a 5 µL aliquot of solutions containing either NMDA (4 mM) or kainate (1 mM) was injected into the vitreous body of one eye of each animal using a Hamilton microsyringe (Hamilton Company, Reno, NV, USA) with a 33-gauge needle, and the other eye was left untreated. Nafamostat (0.4, 2 and 10 nmol/eye), sepimostat (1, 10 and 100 nmol/eye), and other chemicals were premixed with NMDA or kainate solutions in the same amounts as described above and injected into the vitreous body. All injections were performed with the aid of the microscope used for ocular surgery, ensuring no injury to the lens or retina during injection. Two weeks after injections, the animals were euthanised by intraperitoneal injection of an excess dose of pentobarbital. The eyes were enucleated and fixed in a neutral buffered solution containing 10% formaldehyde 24 h at room temperature and processed for histological evaluation as described below.

Retinal ischaemia was induced by the elevation of intraocular pressure. With the animals under halothane anaesthesia, a needle with a polyethylene catheter connected to a reservoir containing sterile isotonic saline was inserted into the anterior chamber of the right eye of each animal. The height of the reservoir was adjusted to maintain 130 mm Hg intraocular pressure for 45 min. The body temperature was kept at 37°C throughout the experiment with a thermal controller unit. Nafamostat (10 nmol/eye) and its vehicle were injected into the vitreous body of the other eye, respectively, and MK-801 (10 mg/kg) was administered intraperitoneally 1 h before the elevation of intraocular pressure. One week after ischaemic insult, the eyes were fixed in the same manner as described above for histological evaluation.

### Histological evaluation

The fixed eyes were rinsed, dehydrated and embedded in paraffin, and 3 µm thickness sections on glass slides were stained with haematoxylin and eosin. Eight cross sections of the retina through the optic disc, taken at 45 µm intervals, were prepared, and three out of these eight sections were randomly selected for histological evaluation. The light microscopic images of the retinas were obtained with a fully automated digital slide scanner (NanoZoomer Digital Pathology™, Hamamatsu Photonics, Sizuoka, Japan). For each image, the IPL thickness was measured and the number of cells in the GCL was determined within an approximately 800 µm expanse of the retina, starting at a distance of 700 µm from the centre of the optic disc. Data from three sections were averaged and used as the representative value for each eye.

### Receptor-binding assay

All binding assay experiments were performed by MDS Pharma Services Ltd. (King of Prussia, PA, USA) in accordance with the procedures described previously^38^. Briefly, cerebral cortices were isolated from the brain of male Wistar-derived rats weighing 175 ± 25 g and homogenised in Tris–HCl buffer (pH 7.4) to prepare *in situ* membrane proteins including glutamate NMDA polyamine receptors. A 5 mg aliquot of the homogenate was incubated with 2 nM [^3^H]ifenprodil in 50 mM Tris–HCl buffer (pH 7.4) for 120 min at 4°C. Test compounds (nafamostat and sepimostat) or the reference compound (ifenprodil) were added to the reaction mixture at the desired concentrations. After the incubation period, the membranes were filtered and washed, and the radioactivity remaining on the filters was measured using a liquid scintillation counter. GBR 12909 (5 µM), which is a sigma receptor antagonist, was used to mask NMDA-unrelated sites^54^. Non-specific binding was determined in the presence of 10 µM ifenprodil. Each displacement curve for ifenprodil binding by the test compounds was fitted with a non-linear least squares regression analysis, and IC_50_ and inhibition constants (Ki) were calculated using the equation presented by Cheng and Prusoff^55^. Hill plots were generated using the displacement curves and the Hill coefficient, defined as the slope of the Hill plot, was calculated to determine whether the test compounds bound to the ifenprodil-binding site.

### Statistical analysis

Each value depicted in the figures represents the mean ± S.E. All statistical analyses were performed using EXSUS software version 8.0.0 (CAC EXICARE Corporation, Tokyo, Japan). Student’s or Aspin–Welch’s t-test was performed to compare the values between two groups. For multiple comparisons, Dunnett’s test or Tukey’s test was used. Differences were assumed to be statistically significant when P <0.05.

## Supplementary information

Supplementary Information.

## Data Availability

All the datasets from the present study may be obtained from the corresponding author upon request.

## References

[1] Otsuki M (2006). Consensus of primary care in acute pancreatitis in Japan. World J. Gastroenterol..

[2] Yokoe M (2015). Japanese guidelines for the management of acute pancreatitis: Japanese Guidelines 2015. J. Hepatobiliary Pancreat. Sci..

[3] Aoyama T (1984). Pharmacological studies of FUT-175, nafamstat mesilate. I. Inhibition of protease activity in *in vitro* and *in vivo* experiments. Jpn. J. Pharmacol..

[4] Chen T (2014). Nafamostat mesilate attenuates neuronal damage in a rat model of transient focal cerebral ischemia through thrombin inhibition. Sci. Rep..

[5] Na KR (2016). Nafamostat Mesilate Attenuates Ischemia-Reperfusion-Induced Renal Injury. Transplant. Proc..

[6] Duan HQ (2018). Nafamostat mesilate attenuates inflammation and apoptosis and promotes locomotor recovery after spinal cord injury. CNS Neurosci. Ther..

[7] Mander S (2018). Nafamostat mesilate negatively regulates the metastasis of triple-negative breast cancer cells. Arch. Pharm. Res..

[8] Yamashita Y (2007). Antitumor effects of Nafamostat mesilate on head and neck squamous cell carcinoma. Auris Nasus Larynx.

[9] Liu Y (2017). Nafamostat Mesilate Improves Neurological Outcome and Axonal Regeneration after Stroke in Rats. Mol. Neurobiol..

[10] J.B. J (2017). Glaucoma. Lancet.

[11] Quigley HA (2011). Glaucoma. Lancet.

[12] Quigley HA, Broman AT (2006). The number of people with glaucoma worldwide in 2010 and 2020. Br. J. Ophthalmol..

[13] Weinreb RN (2007). Glaucoma neuroprotection: What is it? Why is it needed?. Can. J. Ophthalmol..

[14] Adornetto A, Russo R, Parisi V (2019). Neuroinflammation as a target for glaucoma therapy. Neural. Regen. Res..

[15] Doucette LP, Rasnitsyn A, Seifi M, Walter MA (2015). The interactions of genes, age, and environment in glaucoma pathogenesis. Surv. Ophthalmol..

[16] Pinazo-Duran MD, Zanon-Moreno V, Gallego-Pinazo R, Garcia-Medina JJ (2015). Oxidative stress and mitochondrial failure in the pathogenesis of glaucoma neurodegeneration. Prog. Brain Res..

[17] Weinreb RN, Aung T, Medeiros FA (2014). The pathophysiology and treatment of glaucoma: a review. JAMA.

[18] Wiggs JL, Pasquale LR (2017). Genetics of glaucoma. Hum. Mol. Genet..

[19] Nakayama T (1993). Synthesis and structure-activity study of protease inhibitors. V. Chemical modification of 6-amidino-2-naphthyl 4-guanidinobenzoate. Chem. Pharm. Bull. (Tokyo).

[20] Oda M (1990). Pharmacological studies on 6-amidino-2-naphthyl[4-(4,5-dihydro-1H-imidazol-2-yl)amino] benzoate dimethane sulfonate (FUT-187). I: Inhibitory activities on various kinds of enzymes *in vitro* and anticomplement activity *in vivo*. Jpn. J. Pharmacol..

[21] Tsuda Y (2012). Effect of nafamostat on N-methyl-D-aspartate-induced retinal neuronal and capillary degeneration in rats. Biol. Pharm. Bull..

[22] Muir KW, Lees KR (1995). Clinical experience with excitatory amino acid antagonist drugs. Stroke.

[23] Morizane C (1997). N(omega)-nitro-L-arginine methyl ester protects retinal neurons against N-methyl-D-aspartate-induced neurotoxicity *in vivo*. Eur. J. Pharmacol..

[24] Siliprandi R (1992). N-methyl-D-aspartate-induced neurotoxicity in the adult rat retina. Vis. Neurosci..

[25] Tamura Y, Okamura HM, Minato K (1997). Y. Synthetic inhibitors of trypsin, plasmin, kallikrein, thrombin, C1r-, and C1 esterase. Biochim. Biophys. Acta.

[26] Laude A (2010). Intravitreal therapy for neovascular age-related macular degeneration and inter-individual variations in vitreous pharmacokinetics. Prog. Retin. Eye Res..

[27] Reynolds IJ AE (1992). Pentamidine is an N-methyl-D-aspartate receptor antagonist and is neuroprotective *in vitro*. J. Neurosci..

[28] Williams K (1993). Ifenprodil discriminates subtypes of the N-methyl-D-aspartate receptor: selectivity and mechanisms at recombinant heteromeric receptors. Mol. Pharmacol..

[29] Zhang S (2000). Protective effects of ifenprodil against glutamate-induced neurotoxicity in cultured retinal neurons. Graefes Arch. Clin. Exp. Ophthalmol..

[30] Huettner JE, Bean BP (1988). Block of N-methyl-D-aspartate-activated current by the anticonvulsant MK-801: selective binding to open channels. Proc. Natl. Acad. Sci. USA.

[31] Sparapani M, Dall’Olio R, Gandolfi O, Ciani E, Contestabile A (1997). Neurotoxicity of polyamines and pharmacological neuroprotection in cultures of rat cerebellar granule cells. Exp. Neurol..

[32] Freise J, Schmidt FW, Magerstedt P, Schmid K (1985). Gabexate mesilate and camostate: New inhibitors of phospholipase A2 and their influence on the alpha-amylase activity in serum of patients with acute pancreatitis. Clin. Biochem..

[33] Freise J, Wittenberg H, Magerstedt P (1989). *In vitro* inhibition of phospholipase A2 by gabexate mesilate, camostate, and aprotinine. Klin. Wochenschr..

[34] Tamura Y, Hirado M, Okamura K, Minato Y, Fujii S (1977). Synthetic inhibitors of trypsin, plasmin, kallikrein, thrombin, C1r-, and C1 esterase. Biochim. Biophys. Acta.

[35] Kumada MNM, Hara A (2005). Tissue type plasminogen activator facilitates NMDA-receptor-mediated retinal apoptosis through an independent fibrinolytic cascade. Invest. Ophthalmol. Vis. Sci..

[36] Mali RS, Cheng M, Chintala SK (2005). Plasminogen activators promote excitotoxicity-induced retinal damage. FASEB J..

[37] Rock N, Chintala SK (2008). Mechanisms regulating plasminogen activators in transformed retinal ganglion cells. Exp. Eye Res..

[38] Schoemaker H, Allen J, Langer SZ (1990). Binding of [3H]ifenprodil, a novel NMDA antagonist, to a polyamine-sensitive site in the rat cerebral cortex. Eur. J. Pharmacol..

[39] Regan MC, Romero-Hernandez A, Furukawa H (2015). A structural biology perspective on NMDA receptor pharmacology and function. Curr. Opin. Struct. Biol..

[40] Hansen KB (2018). Structure, function, and allosteric modulation of NMDA receptors. J. Gen. Physiol..

[41] Kemp JA, McKernan RM (2002). NMDA receptor pathways as drug targets. Nat. Neurosci..

[42] Perin-Dureau F, Rachline J, Neyton J, Paoletti P (2002). Mapping the binding site of the neuroprotectant ifenprodil on NMDA receptors. J. Neurosci..

[43] Marinelli L (2007). Homology modeling of NR2B modulatory domain of NMDA receptor and analysis of ifenprodil binding. ChemMedChem.

[44] Gallagher MJ, Huang H, Pritchett DB, Lynch DR (1996). Interactions between ifenprodil and the NR2B subunit of the N-methyl-D-aspartate receptor. J. Biol. Chem..

[45] Butler TW (1998). (3R,4S)-3-[4-(4-fluorophenyl)-4-hydroxypiperidin-1-yl]chroman-4,7-diol: a conformationally restricted analogue of the NR2B subtype-selective NMDA antagonist (1S,2S)-1-(4-hydroxyphenyl)-2-(4-hydroxy-4-phenylpiperidino)- 1-propanol. J. Med. Chem..

[46] Tamiz AP (1998). Structure-activity relationships for a series of bis(phenylalkyl)amines: potent subtype-selective inhibitors of N-methyl-D-aspartate receptors. J. Med. Chem..

[47] Chenard BL (1995). (1S,2S)-1-(4-hydroxyphenyl)-2-(4-hydroxy-4-phenylpiperidino)-1-propanol: a potent new neuroprotectant which blocks N-methyl-D-aspartate responses. J. Med. Chem..

[48] Duval DRN, Gauffeny C, Nowicki JP, Scatton B (1992). SL 82.0715, an NMDA antagonist acting at the polyamine site, does not induce neurotoxic effects on rat cortical neurons. Neurosci. Lett..

[49] Gill R (2002). Pharmacological characterization of Ro 63-1908 (1-[2-(4-hydroxy-phenoxy)-ethyl]-4-(4-methyl-benzyl)-piperidin-4-ol), a novel subtype-selective N-methyl-D-aspartate antagonist. J. Pharmacol. Exp. Ther..

[50] Gotti B (1988). and SL 82.0715 as cerebral anti-ischemic agents. I. Evidence for efficacy in models of focal cerebral ischemia. J. Pharmacol. Exp. Ther..

[51] Merchant RE (1999). A double-blind, placebo-controlled study of the safety, tolerability and pharmacokinetics of CP-101,606 in patients with a mild or moderate traumatic brain injury. Ann. N. Y. Acad. Sci..

[52] Abe O (1994). Early phase II study of FUT-187 (sepimostat mesilate) on reflux esophagitis–mainly on postgastrectomy reflux esophagitis. Gan To. Kagaku Ryoho.

[53] Makuuchi H, Kanno K, Mitomi T (1995). Clinical usefulness of FUT-187 (sepimostat mesilate) on reflux esophagitis and gastritis after gastrectomy. Gan To. Kagaku Ryoho.

[54] Hashimoto K, Mantione CR, Spada MR, Neumeyer JL, London ED (1994). Further characterization of [3H]ifenprodil binding in rat brain. European Journal of Pharmacology: Molecular Pharmacology.

[55] Yung-Chi C, Prusoff WH (1973). Relationship between the inhibition constant (KI) and the concentration of inhibitor which causes 50 per cent inhibition (I50) of an enzymatic reaction. Biochem. Pharmacol..

