# Supplementary material for: Nafamostat and sepimostat identified as novel neuroprotective agents via NR2B N-methyl-D-aspartate receptor antagonism using a rat retinal excitotoxicity model
Source: Sci Rep. 2019 Dec 31;9:20409. doi: 10.1038/s41598-019-56905-x (PMC6938488; doi:10.1038/s41598-019-56905-x)
Supplement: Supplementary file 1 — Supplementary Information. [file 41598_2019_56905_MOESM1_ESM.docx]

Nafamostat and sepimostat identified as novel neuroprotective agents via NR2B N-methyl-D-aspartate receptor antagonism using a rat retinal excitotoxicity model

Masahiro Fuwa^1,3^, Masaaki Kageyama^2^, Koji Ohashi^1^, Masaaki Sasaoka^2^, Ryuichi Sato^3^, Masami Tanaka^3^ and Kei Tashiro^3*^

^1^ Research and Development, Santen Pharmaceutical Co., Ltd., Nara, Japan

^2^ Global Alliances and External Research, Santen Pharmaceutical Co., Ltd., Nara, Japan

^3^ Department of Genomic Medical Sciences, Kyoto Prefectural University of Medicine, Kyoto, Japan

^*^Correspondence to:

Kei Tashiro, M.D., Ph.D., Professor, Department of Genomic Medical Sciences, Kyoto Prefectural University of Medicine, Kawaramachi-Hirokoji, Kamigyo-ku Kyoto602-8566, Japan

Tel: +81-75-251-5346, Fax: +81-75-251-5346, E-mail: [tashiro@koto.kpu-m.ac.jp](mailto:tashiro@koto.kpu-m.ac.jp)

**Supplementary Figure S1.** Chemical structures of the serine protease inhibitors: (a) nafamostat, (b) sepimostat, (c) gabexate and (d) camostat, and the N-methyl-D-aspartate (NMDA) receptor antagonist (e) pentamidine. Note that both nafamostat and sepimostat possess an amidinophenyl group in their structure, which is shared by pentamidine but not by camostat or gabexate.

**Supplementary Figure S2.** Schematic diagram of the NMDA receptor complex and potential binding sites of NMDA receptor antagonists tested in this study.

The original drawing provided by Regan, M. C. et al.^39^ and Hansen, K. B. et al.^40^ are simplified. NMDA receptors comprise heterotetramers of NR1 and NR2 subunits forming the ion channel pore structure. The authentic NMDA receptor antagonist MK-801 binds to a site located in the ion channel pore of the NMDA receptor complex, resulting in the blockage of ion influx, including Ca^2+^. The binding sites for the other NMDA receptor antagonists including ifenprodil and nafamostat are located in the extracellular domain of the NR2B subunit. A detailed description of the interaction between the NR2B subunit and the NMDA receptor antagonists is included in the text and Fig. 9.

**Supplementary Figure S3.** Effects of aprotinin on NMDA-induced retinal degeneration in rats

Upper and lower panels show the changes in the ganglion cell layer (GCL) cell number and inner plexiform layer (IPL) thickness, respectively, after intravitreal injections of the vehicle (open column), NMDA alone (closed column, 20 nmol/eye) and NMDA plus aprotinin (AP, grey column, 10 nmol/eye). Each value represents the mean ± S.E. for four to five rats. ***P < 0.001, compared with vehicle; ^#^P < 0.05, compared with NMDA alone by Tukey's multiple comparison test.

**Supplementary Figure S4.** Effects of pentamidine on [3H]ifenprodil-binding in rat cerebral cortical membranes

The binding assay was performed in the presence of 5 µM GBR12909, a sigma receptor antagonist. The closed circles show the percentages of pentamidine inhibition of [^3^H]ifenprodil binding, and the open triangles show the same using unlabelled ifenprodil as a positive control. The IC_50_ values for pentamidine and ifenprodil were 55.6 and 0.0121 µM, the K_i_ values were 51.6 and 0.0112 µM, and the Hill coefficients were 2.11 and 0.664, respectively. Each value represents the mean of two to three replicates.
